# Postpartum Atypical Hemolytic Uremic Syndrome Complicating β-Thalassemia Intermedia: A Case Report and Literature Review

**DOI:** 10.3390/jcm15145740

**Published:** 2026-07-22

**Authors:** Baorong Gao, Yali Miao, Shanza Waseem

**Affiliations:** 1 Key Laboratory of Birth Defects and Related Diseases of Women and Children, Ministry of Education, Sichuan University, Chengdu 610041, China; 2 Department of Obstetrics and Gynaecology, West China Second University Hospital, Chengdu 610041, China

**Keywords:** atypical hemolytic uremic syndrome, thalassemia, pregnancy, postpartum hemorrhage, case report

## Abstract

**Background:** Atypical hemolytic uremic syndrome (aHUS) is a life-threatening thrombotic microangiopathy driven by uncontrolled activation of the complement alternative pathway, with pregnancy triggering 10–20% of cases (pregnancy-associated aHUS, p-aHUS). Thalassemia, a chronic hemolytic anemia, creates a state of subclinical complement dysregulation; however, its clinical association with p-aHUS has rarely been reported. **Methods:** This study presents a 27-year-old primigravida with β-thalassemia intermedia requiring transfusions during pregnancy (CD17 heterozygote) who developed severe postpartum hemorrhage (1760 mL) after vaginal delivery. Over postpartum days 0–4, the patient was monitored for hematological and renal parameters. Laboratory investigations included peripheral smear, ADAMTS13 activity assay, direct Coombs test, and sC5b-9 complement level measurement. **Results:** Over postpartum days 0–4, the patient developed microangiopathic hemolytic anemia (hemoglobin nadir 46 g/L, lactate dehydrogenase peak 3436 U/L), thrombocytopenia (platelet nadir 31 × 10^9^/L), and acute kidney injury (creatinine peak 663 μmol/L). Peripheral smear showed 4% schistocytes. Subsequent detection revealed normal ADAMTS13 activity (90%), negative direct Coombs test, and elevated sC5b-9 (384.95 ng/mL; normal < 340). The diagnosis of p-aHUS was confirmed, and the patient received hemodialysis and recovered within three months. **Conclusions:** These findings suggest that chronic complement dysregulation secondary to thalassemia, combined with pregnancy-related complement stress and postpartum hemorrhage, may contribute to the development of p-aHUS. Therefore, thalassemia should be considered a potential sensitizing condition for p-aHUS, warranting close monitoring and complement evaluation in such patients.

## 1. Introduction

Atypical hemolytic uremic syndrome (aHUS) is a rare but severe thrombotic microangiopathy (TMA) with an estimated annual incidence of 0.23 to 1.9 per million population and a prevalence of 2.2 to 9.4 per million among individuals aged 20 years or younger [1,2]. The disorder is characterized by the clinical triad of microangiopathic hemolytic anemia, thrombocytopenia, and acute kidney injury (AKI), driven by uncontrolled activation of the complement alternative pathway [3,4].

Pregnancy is a well-recognized trigger of aHUS, accounting for approximately 10–20% of all cases, and pregnancy-associated aHUS (p-aHUS) often occurs in the postpartum period [5,6]—a pattern that reflects the unique physiological complement stress test imposed by pregnancy, with peripartum complications such as postpartum hemorrhage, preeclampsia, and HELLP syndrome serving as additional drivers of uncontrolled complement activation, further exacerbating p-aHUS [7,8].

β-Thalassemia is an inherited disorder of hemoglobin production that causes chronic hemolytic anemia and secondary iron overload [9,10]. Growing evidence redefines this condition as a systemic vasculopathy characterized by chronic hypercoagulability, endothelial dysfunction, and ongoing inflammation [11,12]. In addition, patients with β-thalassemia often show reduced levels of protective proteins CD55 and CD59 on erythrocytes, together with deposits of the membrane attack complex C5b-9 on circulating cells [13,14]. This creates a primed system vulnerable to a second trigger that can precipitate uncontrolled TMA. Mutations in complement regulatory genes act as a first hit, while added stressors such as infection, pregnancy, or hemolysis amplify complement activation beyond the body’s normal control mechanisms [15,16].

Although a mechanistic link between thalassemia and aHUS is plausible, direct clinical evidence remains exceptionally scarce. A systematic search of PubMed and Embase through 20 May 2026, using the terms “thalassemia” AND “atypical hemolytic uremic syndrome” yielded only three relevant publications. These comprise a case of a 21-month-old girl with thalassemia and aHUS complicated by peripheral gangrene [17], a report of pregnancy-associated aHUS in a thalassemia patient with a complement factor H (CFH) mutation who was treated with eculizumab and luspatercept [15], and a child with β-thalassemia who developed aHUS following gastroenteritis; in this last case, the absence of complement studies left the underlying mechanism uncharacterized [15,18].

Additional observations reinforce the notion that complement dysregulation alone is insufficient to cause aHUS. An asymptomatic α-thalassemia carrier harboring a CFH mutation never developed the disease, illustrating the requirement for a second trigger [19,20]. Furthermore, in a cohort of 47 aHUS cases, two patients had thalassemia, yet complement mutation profiling, postpartum hemorrhage status, and transfusion requirements were not reported for these individuals [15,17]. The absence of data from these patients precludes any mechanistic insight into the interplay between thalassemia-related hemolysis and complement dysregulation, highlighting a critical gap in the literature [21,22]. Here, we report a detailed case of postpartum aHUS in a woman with β-thalassemia intermedia and propose a mechanistic “three-hit” model.

## 2. Case Presentation

A 27-year-old primigravida of Asian descent with β-thalassemia intermedia (CD17 [A>T] heterozygous mutation in the β-globin gene) was followed in our clinic. The patient had not undergone splenectomy. She required packed red blood cell (pRBC) transfusions every 4–6 weeks during pregnancy to maintain her hemoglobin above 90 g/L. Iron chelation with deferasirox had been discontinued 3 months before conception. Pre-conception laboratory values included hemoglobin 116 g/L following transfusion, platelets 177 × 10^9^/L, serum creatinine 48 μmol/L, and lactate dehydrogenase (LDH) 178 U/L. Serum ferritin at that time was 23 ng/mL, reflecting effective pre-conception chelation therapy rather than indicating iron overload. The patient reported no family history of aHUS, thrombotic microangiopathy, complement disorders, chronic kidney disease, or unexplained hemolytic anemia.

At 40^+1^ weeks’ gestation, she had a spontaneous vaginal delivery, and 100 μg of intravenous carbetocin was given for routine active management. Fifteen minutes later, the placenta was delivered intact manually. Uterine atony of the lower segment and a 5-cm right vaginal wall hematoma were identified. Following hematoma evacuation and hemostasis, significant bleeding recurred, with uterine atony unresponsive to standard uterotonics. Blood loss at 2 h postpartum was 1760 mL and she received 4 g of fibrinogen concentrate, 600 mL of fresh frozen plasma (FFP), and 3 units of pRBCs.

**Figure 1:**
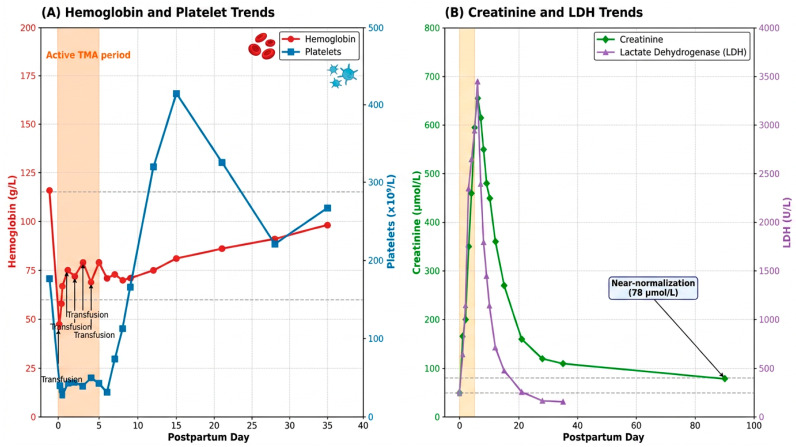
Longitudinal trends of key laboratory parameters. (**A**) Hemoglobin (red) and platelet (blue) trends. (**B**) Creatinine (green) and lactate dehydrogenase (LDH, purple) trends. The orange shaded area indicates the period of acute illness (postpartum days 0–5). The dashed line represents the day of hemodialysis initiation (postpartum day 5).

Between 2 and 5 h postpartum, urine output fell to 25 mL and was unresponsive to furosemide, while serum creatinine and LDH levels rose progressively. Over the subsequent four days, despite cumulative transfusions of 10.5 units of pRBCs, 1050 mL of FFP, and 2 units of platelets, the patient’s hemoglobin fluctuated between 46 and 79 g/L and her platelet count remained persistently low (28–50 × 10^9^/L). Concurrently, by postpartum day 5, creatinine had risen to 663 μmol/L and LDH peaked at 3436 U/L. Details are shown in Table 1 and Figure 1.

A peripheral blood smear revealed 4% schistocytes. The diagnostic workup demonstrated a normal ADAMTS13 activity (90%), a negative direct Coombs test, negative antiphospholipid antibodies, and an elevated sC5b-9 level of 384.95 ng/mL (normal < 340 ng/mL), indicative of terminal complement pathway activation. These findings were indicative of p-aHUS.

Following diagnosis, the patient was treated with hemodialysis (initiated on postpartum day 5) and supportive care, as complement-targeted therapy with eculizumab was not available at our institution. Renal function and platelet count gradually improved, and she was discharged on postpartum day 15. At three-month follow-up, her serum creatinine had normalized to 78 μmol/L.

Written informed consent was obtained from the patient, and the requirement for formal ethical approval was waived by the hospital ethics committee for this case report.

## 3. Discussion

This case detailed p-aHUS presentation in a patient with β-thalassemia intermedia, distinguished by three key features: (1) direct documentation of terminal complement activation (sC5b-9 384.95 ng/mL); (2) complete transfusion-refractory cytopenia data during acute TMA; and (3) a severe postpartum hemorrhage as a likely amplifying trigger. These findings indicate that thalassemia is not an incidental comorbidity but a complement-sensitizing condition that, when compounded by pregnancy, can precipitate decompensation.

### 3.1. What the Literature Has Established and the Questions That Remain

Prior reports have suggested a potential link between thalassemia and aHUS, but the mechanistic evidence has remained indirect. The strongest prior evidence came from a pregnancy-associated aHUS case in a thalassemia patient with a CFH mutation, leading to the proposal of a “two-hit” model in which hemolysis-driven complement activation acts on a genetically susceptible background [16]. A contrasting case of an asymptomatic CFH mutation carrier with thalassemia trait who never developed disease reinforced the concept that an additional precipitating event is required to overcome complement tolerance [16,20,21]. Crucially, none of these earlier reports incorporated direct measurement of terminal complement complex (sC5b-9) during the acute phase or systematically documented transfusion-refractory cytopenias. Our case addresses these gaps, providing the first direct evidence of terminal pathway activation and illustrating how severe postpartum hemorrhage can serve as the critical third hit that transforms a chronic hemolytic predisposition into fulminant p-aHUS.

### 3.2. A Three-Hit Model for p-aHUS in β-Thalassemia

**Figure 2:**
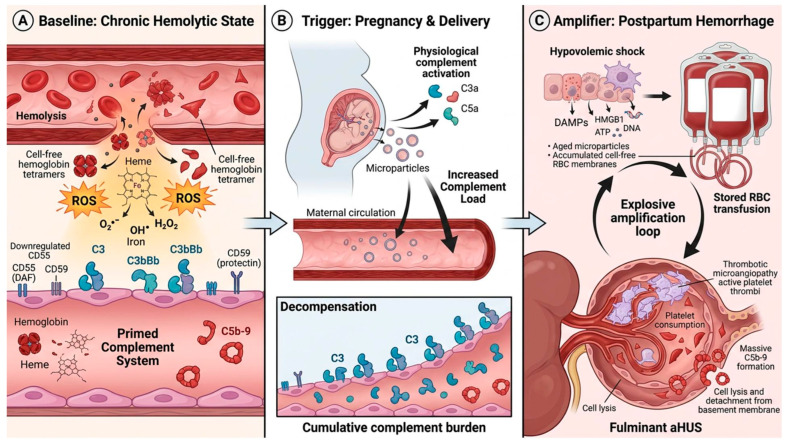
Three-hit mechanistic model of pregnancy-associated atypical hemolytic uremic syndrome (p-aHUS) in β-thalassemia. Abbreviations: C5b-9, membrane attack complex; DAMPs, damage-associated molecular patterns; ROS, reactive oxygen species.

We propose a three-hit pathophysiological model that mirrors our patient’s clinical course (Figure 2).

**Hit** **1:** *Baseline susceptibility—chronic complement dysregulation in thalassemia.*

First, persistent intravascular hemolysis releases cell-free hemoglobin and heme [18]. Second, free heme dose-dependently inhibits factor I-mediated degradation of C3b, disabling the key regulatory step that normally dismantles the C3 convertase and resulting in unchecked complement amplification [19]. Third, iron overload generates reactive oxygen species (ROS) that downregulate the membrane-bound complement regulatory proteins CD55 and CD59. In β-thalassemia major patients, CD55 expression on red blood cells is significantly reduced [22]. The net effect is a “compensated complementopathy”—a primed system held in check by residual regulatory capacity but vulnerable to decompensation from a second trigger [23,24]. Notably, most thalassemia patients never develop aHUS, because thalassemia alone merely establishes a baseline state of chronic alternative pathway activation that is insufficient to trigger clinical disease.

**Hit** **2:** *Trigger—pregnancy and delivery as a complement stress test.*

Normal pregnancy induces progressive complement activation, with C3a, C5a, and sC5b-9 levels rising toward term [25,26]. At delivery, placental separation releases trophoblast-derived microparticles directly into the maternal circulation, activating both complement and coagulation cascades [27,28]. In our patient, this physiological complement load overwhelmed her already primed system. The fact that 79–94% of p-aHUS cases present postpartum rather than antenatally reflects exactly this timing: the peak complement challenge occurs at placental separation [5,6,29].

**Hit** **3:** *Amplifier—postpartum hemorrhage, shock, and massive transfusion.*

This is where our patient’s case diverges from previously reported thalassemia-associated aHUS. Her severe PPH (1760 mL) and subsequent resuscitation provided a third, amplifying hit that converted subclinical complement activation into fulminant, self-sustaining TMA. Three amplifier mechanisms converged. First, hypovolemic shock from PPH caused renal ischemia–reperfusion injury, releasing damage-associated molecular patterns (DAMPs) [30,31]. Second, massive transfusion (10.5 units pRBCs, 1050 mL FFP, 2 units platelets) introduced stored blood components that bear C3 fragments and cell-free hemoglobin, fueling complement activation [32,33]. Third, the thalassemia-associated acquired platelet storage pool defect—chronic in vivo platelet pre-activation leading to partial degranulation and “exhausted” platelets—both predisposed her to PPH and impaired hemostatic recovery [34,35,36,37,38,39].

### 3.3. Why This Case Was Diagnostically Challenging and What Clues Point to p-aHUS

Three features in our patient provided critical diagnostic clues that distinguished p-aHUS from mimics (Table 2) [38,39,40,41,42,43,44,45,46,47,48,49,50]. First, transfusion-refractory thrombocytopenia (platelet nadir 28 × 10^9^/L despite 2 units of platelets) indicated consumption rather than dilution. Second, LDH elevation out of proportion to measured blood loss signaled intravascular hemolysis beyond what PPH alone could explain. Third, schistocytes on peripheral smear (4%)-the presence of >1% schistocytes has a sensitivity of 95% and a specificity of 98% for TMA [51,52].

Importantly, p-aHUS was distinguished from thrombotic thrombocytopenic purpura (TTP) by a normal ADAMTS13 level (90%) and from HELLP syndrome by the absence of significant transaminitis and the timing of platelet recovery (HELLP typically improves within 48–72 h postpartum [42,43], whereas our patient’s thrombocytopenia persisted through day 4). The elevated sC5b-9 provided confirmatory evidence of terminal complement pathway activation, establishing p-aHUS as the diagnosis [49,53].

### 3.4. Therapeutic Implications and Future Pregnancy Counseling

Our patient received supportive care with hemodialysis but did not receive eculizumab owing to limited drug availability. Although she achieved favorable renal recovery (creatinine 78 μmol/L at 3 months), this outcome should not be expected routinely. Multiple complement inhibitors have been extensively studied and are now available in clinical practice [50,54,55,56,57,58,59,60,61,62,63,64,65,66]. These agents can be categorized into two groups based on their molecular structure and target: monoclonal antibodies targeting C5, and peptide-based inhibitors.

Among the C5-targeting monoclonal antibodies, eculizumab and ravulizmab are the only agents with robust clinical evidence and regulatory approval specifically for aHUS [54,55,56,57,60,61]. Both share the same mechanism of action, but ravulizumab has a longer half-life that allows maintenance dosing every 8 weeks instead of every 2 weeks, significantly reducing treatment burden [60,61]. Pozelimab, a fully human monoclonal anti-C5 antibody administered subcutaneously, offers the convenience of self-administration but has limited experience in aHUS and lacks pregnancy safety data [64].

In contrast, zilucoplan and pegcetacoplan are peptide-based inhibitors. Zilucoplan is a subcutaneously administered C5 inhibitor approved for myasthenia gravis, while pegcetacoplan targets C3/C3b and is approved for paroxysmal nocturnal hemoglobinuria [62,65]. Although both have attractive subcutaneous routes, their role in aHUS remains investigational, and data in pregnancy are lacking [63,66,67].

Clinicians encountering similar cases should not delay complement blockade while awaiting genetic testing results; the diagnosis of p-aHUS is clinical and can be supported by an elevated sC5b-9 level, which is available within hours at many centers [49,50,53]. Treatment should continue for a minimum of 6–12 months postpartum, given the elevated risk of complement dysregulation and disease relapse during this period [5,61]. However, all C5 and C3 inhibitors carry a 1000- to 2000-fold increased risk of meningococcal disease, necessitating vaccination against Neisseria meningitidis at least two weeks prior to treatment initiation [68,69]. For C3 inhibitors such as pegcetacoplan, vaccination against Streptococcus pneumoniae is also recommended [67].

Given the rarity of aHUS, universal screening of all thalassemia patients is not feasible; however, closer surveillance should be reserved for those with a family history of aHUS or unexplained TMA, prior unexplained hemolysis or thrombosis, or known complement gene mutations. Whether prophylactic eculizumab or ravulizumab should be considered for thalassemia patients suspected of having aHUS requires further study and is not currently standard of care [70].

Future pregnancy carries a 20–50% risk of p-aHUS recurrence and is strongly associated with underlying complement gene mutations [6]. Although we did not perform complement genetic testing in our patient due to resource limitations, we strongly recommend it before any future pregnancy. Pathogenic variants in CFH, CFI, MCP/CD46, C3, CFB, and THBD are identified in approximately 40–60% of aHUS cases [70], and 12% of patients carry mutations in more than one gene, mandating comprehensive multi-gene panel testing [71].

## 4. Limitations

This case report has several limitations. First, as a single case, it cannot establish causation; the observed association between thalassemia and p-aHUS may be coincidental, although the mechanistic plausibility is strong. Second, complement genetic testing was not performed, so we cannot determine whether our patient carried an inherited complement mutation (e.g., in CFH, CFI, or MCP) that acted synergistically with thalassemia to precipitate disease. Third, eculizumab was not administered, limiting assessment of therapeutic response. Fourth, the classification of this patient’s thalassemia as intermedia rather than major should be noted, and this phenotypic distinction may affect the generalizability of our findings to patients with transfusion-dependent thalassemia major. Despite these limitations, this report represents the first detailed, complement-profiled case of p-aHUS in a thalassemia patient and provides hypothesis-generating evidence for future prospective studies.

## 5. Conclusions

This first complement-profiled case demonstrates that β-thalassemia intermedia can act as a sensitizing condition for p-aHUS through a three-hit mechanism. Future studies should evaluate the incidence of aHUS in pregnant thalassemia populations and identify complement-modifying genetic variants. Clinicians should maintain a high index of suspicion for p-aHUS in thalassemia patients presenting with postpartum thrombocytopenia, hemolytic anemia, and acute kidney injury.

## Figures and Tables

**Table 1:** Laboratory parameters from admission through follow-up.

Postpartum Time	Cr (μmol/L)	LDH (U/L)	HGB (g/L)	PLT (×10^9^/L)	SF (ng/dL)	Urine Volume (mL)
Prenatal baseline	48	178	116	177	23	—
Postpartum 5 h	—	—	46	40	—	25
Postpartum 9 h	167	620	58	34	—	—
Day 1 (12 h)	199	642	67	28	—	—
Day 1 (28 h)	354	—	75	43	1550.4	92
Day 2	461	2476	72	44	—	1899
Day 3	593	2651	79	39	—	2720
Day 4	652	2935	69	50	—	3990
Day 5	663	3436	79	43	4050	—
Day 7	548	1800	73	74	—	2190
Day 9	450	1173	71	166	976.9	2145
Day 15 (discharge)	267	498	81	414	772.9	1950
3 months	78	—	—	—	254	—

Abbreviations: Cr: creatinine (normal 44–80 μmol/L), LDH: lactate dehydrogenase (normal 120–250 U/L), HGB: hemoglobin (normal 115–155 g/L), PLT: platelets (normal 150–450 × 10^9^/L), SF: serum ferritin (normal 15–150 ng/dL).

**Table 2:** Differential diagnosis of postpartum thrombotic microangiopathy.

Condition	Key Features	Tests	Timing	Therapy
Severe PPH alone	Anemia proportionate to blood loss; platelets normal or mildly decreased; AKI prerenal [38,39]	No schistocytes; LDH normal or mildly elevated (<1.5 × ULN) [38,39]	Immediate postpartum (0–24 h) [38,39]	Volume repletion; transfusion; uterine hemostatic measures [38,39]
HELLP Syndrome	Hemolysis, elevated liver enzymes (AST/ALT > 2 × ULN), low platelets (<100,000/mm^3^); hypertension, proteinuria, epigastric/RUQ pain [40]	Schistocytes on peripheral smear; LDH > 600 IU/L; total bilirubin > 1.2 mg/dL; platelets recover 48–72 h postpartum [40]	Antenatal (70%) or ≤48 h postpartum (30%); peaks 24 h after delivery [41]	Delivery (definitive); magnesium sulfate (seizure prophylaxis); antihypertensives (IV labetalol, hydralazine, or nifedipine); corticosteroids if <34 weeks for fetal lung maturity; platelets/FFP if DIC [42,43]
Thrombotic Thrombocytopenic Purpura (TTP)	ADAMTS13 activity < 10%; neurological symptoms; fever [44]	Schistocytes on blood smear; ADAMTS13 activity < 10% [45]	Any time	Plasma exchange (first-line); corticosteroids; rituximab (refractory/relapsing cases) [44,46]
p-aHUS (pregnancy-associated atypical hemolytic uremic syndrome)	ADAMTS13 normal; sC5b-9 elevated; severe AKI [47]	Schistocytes 1–5%; sC5b-9 > 340 ng/mL; complement gene mutation in ~60% [47]	24 h–4 weeks postpartum	Eculizumab (anti-C5); plasma exchange if eculizumab unavailable [48,49,50]

Abbreviations: ADAMTS13, A Disintegrin And Metalloproteinase with Thrombospondin type 1 motif, member 13; aHUS, atypical hemolytic uremic syndrome; AKI, acute kidney injury; ALT, alanine aminotransferase; AST, aspartate aminotransferase; CD, cluster of differentiation; CFH, complement factor H; Cr, creatinine; DIC, disseminated intravascular coagulation; FFP, fresh frozen plasma; HELLP, hemolysis, elevated liver enzymes, low platelets; HGB, hemoglobin; LDH, lactate dehydrogenase; p-aHUS, pregnancy-associated atypical hemolytic uremic syndrome; PPH, postpartum hemorrhage; PLT, platelets; pRBC, packed red blood cell; RUQ, right upper quadrant; sC5b-9, soluble complement complex C5b-9 (terminal complement complex); SF, serum ferritin; TCC, terminal complement complex; TMA, thrombotic microangiopathy; TTP, thrombotic thrombocytopenic purpura; ULN, upper limit of normal.

## Data Availability

No new data were created or analyzed in this study. Data sharing is not applicable to this article.

## Abbreviations

AKI	Acute kidney injury
aHUS	Atypical hemolytic uremic syndrome
FFP	Fresh frozen plasma
LDH	Lactate dehydrogenase
p-aHUS	Pregnancy-associated atypical hemolytic uremic syndrome
PPH	Postpartum hemorrhage
pRBC	Packed red blood cells
TMA	Thrombotic microangiopathy
TTP	Thrombotic thrombocytopenic purpura
